# Innovative “Green” and Novel Strategies for the Extraction of Bioactive Added Value Compounds from Citrus Wastes—A Review

**DOI:** 10.3390/molecules22050680

**Published:** 2017-04-27

**Authors:** Predrag Putnik, Danijela Bursać Kovačević, Anet Režek Jambrak, Francisco J. Barba, Giancarlo Cravotto, Arianna Binello, Jose Manuel Lorenzo, Avi Shpigelman

**Affiliations:** 1 Faculty of Food Technology and Biotechnology, University of Zagreb, Pierottijeva 6, 10000 Zagreb, Croatia; pputnik@alumni.uconn.edu (P.P.); dbursac@pbf.hr (D.B.K.); arezek@pbf.hr (A.R.J.); 2 Nutrition and Food Science Area, Preventive Medicine and Public Health, Food Science, Toxicology and Forensic Medicine Department, Faculty of Pharmacy, Universitat de València, Avda. Vicent Andrés Estellés, s/n, 46100 Burjassot, Spain; francisco.barba@uv.es; 3 Dipartimento di Scienza e Tecnologia del Farmaco, University of Turin, Via P. Giuria 9, Turin 10125, Italy; arianna.binello@unito.it; 4 Centro Tecnológico de la Carne de Galicia, c/Galicia, 4, San Ciprián de Viñas, 32900 Ourense, Spain; jmlorenzo@ceteca.net; 5 Faculty of Biotechnology and Food Engineering, Technion, Israel Institute of Technology, Haifa 3200003, Israel; avis@bfe.technion.ac.il

**Keywords:** citrus wastes, ultrasound, pulsed electric fields, microwaves, high pressure, supercritical CO_2_

## Abstract

Citrus is a major processed crop that results in large quantities of wastes and by-products rich in various bioactive compounds such as pectins, water soluble and insoluble antioxidants and essential oils. While some of those wastes are currently valorised by various technologies (yet most are discarded or used for feed), effective, non-toxic and profitable extraction strategies could further significantly promote the valorisation and provide both increased profits and high quality bioactives. The present review will describe and summarize the latest works concerning novel and greener methods for valorisation of citrus by-products. The outcomes and effectiveness of those technologies such as microwaves, ultrasound, pulsed electric fields and high pressure is compared both to conventional valorisation technologies and between the novel technologies themselves in order to highlight the advantages and potential scalability of these so-called “enabling technologies”. In many cases the reported novel technologies can enable a valorisation extraction process that is “greener” compared to the conventional technique due to a lower energy consumption and reduced utilization of toxic solvents.

## 1. Introduction

The processing of fruits, such as bananas, mangoes and citrus generates significant amounts of by-products often containing valuable compounds in their peels, pulp and seeds. These by-products pose a complex waste-disposal problem and additional economic burdens on production. Citruses are the world’s most abundant fruit crop with an estimated annual production of 115.5 million tons in 2012 [1]. Oranges (*Citrus × sinensis*) are the most produced citrus fruit (70.6 million ton), followed by mandarins (*Citrus reticulata*) (25.5 million tons), lemons (*Citrus limon*) and limes (several species) (12.9 million tons), and grapefruit (*Citrus × paradisi*) (6.4 million tons) [1]. In 2007 it was reported that around 33% of the total production is industrially processed [2], resulting in 15 million tons of waste per year (2007 figures), which can be estimated to account for 50% of the original processed whole fruit mass [3]. This waste is made up of valuable by-products that can be used for animal feed (for example for ruminants [4], poultry [5] and pigs [6]), the extraction of valuable components and food products. The processing of citrus waste for the recovery of natural value-added compounds, such as fibre [7,8], bioactive compounds (like flavonoids [9]), additives and colorants [10] has increased thanks to consumer demand for non-synthetic and more natural food raw materials [11]. In a sustainable approach, by-products are valued sources of various nutrients and in some cases (for example when no refining step is used) waste-management is cheaper and more efficient [12].

Wastes and by-products from citrus fruits contain large amounts of high-added value compounds and show a variety of valuable opportunities in the technological and health promoting domains. Amongst the available biologically active compounds (BAC) in the citrus by-products, we can consider polyphenols, carotenoids and essential oils (EOs). The polyphenols and carotenoids are known to have numerous health benefits, mostly attributed to their antioxidant activity [13,14]. Polyphenols have significant potential as a lucrative raw material for the production of functional foods, pharmaceuticals and cosmetics. A variety of health benefits, such as anti-carcinogenicity, anti-mutagenicity, anti-allergenicity and antiaging activity [15] have been reported for polyphenols, while their stability during processing has also been studied [16,17,18]. It is important to note that the total content of polyphenols is higher in citrus skin, which is commonly discarded, than in the peeled fruit itself [14,19]. EOs are also common in the various fruit parts yet especially true for the peel, which is a natural source of volatile substances that have recently attracted lots of interest from scientific community. EOs are mixtures of many compounds and consist mainly of isoprenoids, monoterpenes and sesquiterpenes. These BAC are responsible for the scent of many aromatic plants [20,21] and can be used for the manufacture of pharmaceuticals, food flavour additives, natural antimicrobials and for the personal care industries [22]. The use of oils in antimicrobial protection has shown growing demand in industry (e.g., fresh-cut) due to consumer demand for more natural products [23]. Numerous EOs have shown biocidal effects on bacteria, fungi, viruses, protozoa, insects and plants. In fact, in Florida (USA) a Research Laboratory of Citrus and Subtropical Products was founded and one of its missions is the development of new approaches for the conversion of fruit processing wastes into value- added products [24]. For instance, it is considered that the use of waste from the USA citrus industry alone could supply up to 10,000 tons of hesperidin (a polyphenol) per year [19]. The use of by-products therefore shows sustainable environmental benefits combined with increased economic gains as well as the production of nutritious foods that will improve the lives of consumers [3].

One of the main reasons for the low levels of the citrus agroindustry residues utilization is the lack of effective and cost-effective extraction methods for compounds with the required quality. Furthermore, the solvents used for the extraction of BACs from plants are often toxic. This is especially true for the extraction of lipophilic components, for which it is necessary to use organic solvents, such as hexane, petroleum ether, diethyl ether etc. “Green-extractions” therefore have the potential to overcome such limitations and provide higher yields and energy savings [25]. Among the noteworthy “green” solvents used are water and supercritical fluids (such as carbon-dioxide), in addition to renewable solvents (bio-solvents such as ethanol and isopropanol) and some ionic liquids [26]. Yet it is important to note that controversy exists regarding how “green” ionic liquids are due to potential hazards to the environmental eco-system [27].

Market trends are directed towards the development of low-cost foods, Generally Recognized as Safe (GRAS) additives, (natural antioxidants) usually derived from plants (and from (micro)algae), and the by-products remaining after production [28,29]. Previous publications and reviews discussed the importance of utilization and valorisation of food waste in general [30], and specifically the opportunities in valorisation of citrus waste [10,31,32,33], in addition to reviews regarding green and alternative methods for valorisation of waste from other agro-industries [34,35,36,37]. On the other hand, an in-depth review regarding “green” and novel strategies for extraction of added value compounds from citrus wastes is lacking. Therefore, the presented review will summarize state of the art literature regarding “green” and novel strategies for the valorisation of citrus wastes by extraction of beneficial compounds. The summarized knowledge in the review can advance greener and more eco-friendly utilization of citrus by-product for the production of various nutraceuticals, bio-preservatives and functional products.

## 2. Nutritional and Bioactive Composition of Citrus Wastes

**Figure 1:**
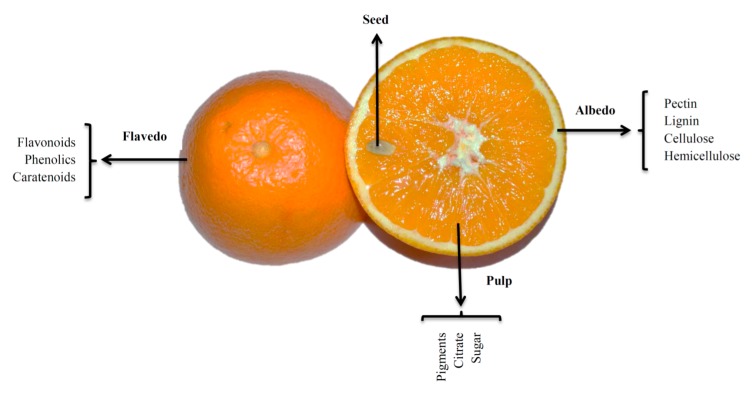
Anatomy of a citrus fruit.

Citrus fruits are commonly processed into cloudy juices [38], and approximately 45 to 60% of the weight of these fruits is discarded as waste, consisting of peel, membrane, juice vesicles and seeds. Citrus peels are subdivided into the epicarp (or flavedo in citrus fruits, which is the coloured peripheral surfaces) and mesocarp (or albedo, the white soft middle layers) as shown in Figure 1. In addition, sugars, EOs and limonoids (a group of highly oxygenated triterpenoids common to the Rutaceae and Meliaceae families [39]) are typical value-added by-products extracted for various industries, but polyphenols and particularly flavonoids are left in the peel, which is then dried, mixed with dried pulps and used for feed [40]. Obtaining such valued by-products from citrus waste may increase the economic yield of the citrus processing industries. In a study, by Sharma et al. [10], the authors observed the use of citrus peel as an economically valuable source of high-added value compounds as it contains a significant amounts of various flavonoids, carotenoids, dietary fibre, sugars, polyphenols, essential oils and ascorbic acid. Citrus waste also contains high levels of sugars suitable for fermentation in bioethanol production [41] and as a substrate for solid state fermentation [42]. The authors reviewed various popular extraction methods for value-added products from citrus waste/by-products and their potential utilization as a source of a number of functional compounds [10].

### 2.1. Dietary Fibre

Citrus fruits are excellent sources of dietary fibre that can be divided into soluble and insoluble fractions. Soluble fibre includes pectin, gum, mucus and a part of cellulose, whereas insoluble dietary fibre mostly includes cellulose, hemicellulose and lignin (Table 1). Gorinstein et al. [7] found that lemon (both peels and peeled fruit) possess the highest antioxidant potential among the citrus fruits studied (lemon, orange and grapefruit) and the peels contain the most fibre (soluble and insoluble) with minimal difference in total fiber content between the peels of those fruits (~14 g/100 g) [7]. On the other hand, as can be seen in Table 1, others suggested a variety dependent difference in the content of fiber. Other studies reported that the dietary fibre represents between 14 g/100 g DM and 57 g/100 g DM, for lemons and orange peels, respectively [7,43]. Generally, citrus peel contains of approximately 50–60% cellulose and hemicellulose, which makes it a good raw material for their extraction. Marín et al. [3] have extracted fibre from lemon and orange peels and determined that pectin, lignin, celluloses and hemicellulose ranged from 13.00–23.03 g/100 g DM, 7.52–7.56 g/100 g DM, 23.06–37.08 g/100 g DM and 8.09–11.04 g/100 g DM of lemon and orange peels, respectively (Table 1). Citrus fibre can be considered as a BAC due to the presence, in addition to the polysaccharides, of polyphenol-like components that can be used as effective inhibitors of lipid oxidation in meat products, thereby improving overall oxidative stability and prolonging the shelf-life of meats [44,45]. In addition, orange juice fibre (peel, pulp and seeds) have been used as a novel fat replacer in ice cream, possibly due to its water and oil retention capabilities [46]. A possible additional application could be as fat replacers in the meat industry that was previously suggested for other fiber sources [47].

#### Pectin

Pectin, represents a family of heterogeneous polysaccharides, made mostly (>65%, in commercial products) of linear α(1→4)-linked d-galacturonic acid (GalA) units, is a natural multifunctional ingredient which imparts textural and rheological properties to a wide range of foods [48]. In 2015, pectin production exceed 60,000 tons worldwide, making it a 1 billion US dollar market [49]. Pectin is used as a thickener, texturizer, emulsifier and stabilizer [48,50], as well as being a component in fillings, confectionary, dietary fibre supplements [51], and drug delivery formulations [52]. It is a natural gelling agent and is particularly used in the production of jams and jellies [53], where it can stabilize other BAC [17]. Commercially, it is extracted as a white to light brown powder, mostly from citrus peels (lime, lemon, orange and grapefruit), apple pomace [53], and sugar beet [54], but quantities vary with plant source. Previous works reported various values of pectin content in citrus waste: 25.00 ± 1.20% DM in citrus waste from the production of orange Brämhults juice [55], 12.07–23.02% DM (Table 1 [3]) for lemon and orange peels respectively, 22.6% DM for Kinnow mandarin waste, 14.2% DM for Mandarin peel waste, 16.1% DM for grapefruit peels [31]. Those results clearly show the opportunities in valorisation of citrus waste for pectin production. In addition to its techno-functional properties in numerous products as described above pectin can be considered as a bioactive compound due to its function as a dietary fibre and more recently reported possible anti-cancer activity originating from its neutral sugar side chains [56].

### 2.2. Polyphenols

**Figure 2:**
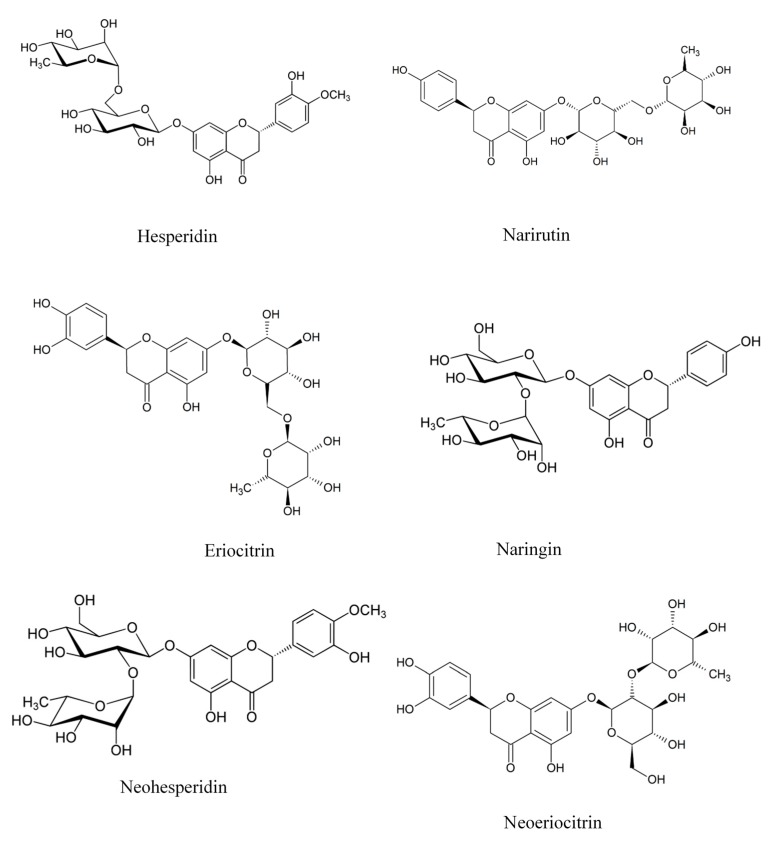
The most abundant flavonoids found in *citrus* species.

Citrus peels are rich in natural flavonoids and contain higher amounts of (poly)phenolics than their edible parts [7]. Flavonoids from peels can be divided into six different groups according to their structure: flavones, flavanones, flavonols, isoflavones, anthocyanidins and flavanols [57]. The chemical structures of the most abundant flavonoids in citrus species are shown on Figure 2. Flavanone and total polyphenols were suggested to account for 2–3% and 0.91–4.92% of dry citrus peels, respectively [58]. In addition small phenolic acids like caffeic, chlorogenic, ferulic, sinapic and *p*-coumaric were also reported in peels [59]. Neoeriocitrin, naringin and neohesperidin are the main flavanones found in the peels of bergamote, lemon and orange with values 400–1000 mg/100 g peel for bergamote, 400–600 mg/100 g peel for lemon peel and 380–1100 mg/ 100 g peel for the peels of sour orange [60,61]. On the other hand, hesperidin and narirutin are the most abundant flavonoids in sweet orange (270–350 mg/100 g dry peel) [62], whereas naringin is the most abundant in grapefruit and bitter orange peels (1400 mg/100 g peel) [63].

### 2.3. Natural Pigments

The pigments derived from citrus peel can be a valuable source of colorants and can replace synthetic pigments. Peel contains two types of natural pigments with differing polarities; one is lipid-soluble carotenoids and the other water-soluble yellow pigments. The most abundant carotenoids in peels are α-carotene, β-carotene, lutein, zeaxanthin and β-cryptoxanthin [64] (Table 2), with total carotenoid values (as β-carotene equivalents) ranging from 11–204 mg/100 g dry peel depending on the citrus fruit variety with *C. reticulata* Blanco contacting the highest values while lemon the lowest [59] among the studied fruits. The most common carotenoid in citrus peel in fruits studied in Taiwan was usually β-carotene [59].

### 2.4. Essential Oils (EOs)

Essential oils (EOs) are a group of volatile aromatic compounds produced by several plant species. These compounds have been used since ancient times as flavouring agents for the preparation of food, medicine, pharmaceuticals and cosmetic products [65,66,67,68]. EOs have beneficial properties on general health [69,70,71,72,73], as well as displaying antibacterial, antifungal and insecticidal properties [65,74,75]. EOs are lipophilic molecules, liquid at room temperature, with low aqueous solubility [76]. The main constituents found in essential oils usually include alkenes, acids, alcohols, aldehydes, esters, ketones, phenols, and nitrogenated compounds [77].

## 3. Valorisation of Citrus Waste and By-products by Extraction of BACs Using Novel Strategies

### 3.1. Pectins

Large structural diversity exists among pectins, not only due to different plant origins but also due to the used extraction process. High-viscosity industrial pectins are usually highly esterified (e.g., methoxy, 8–12%), have an average molecular weight of 100–300 kDa and a high GalA ≥65% content [78]. The current industrial extraction of pectins from dried peels generally employs mineral acids to lower the pH (1.3–3) at T = 60–100 °C, while the duration of the process ranges from 20–360 min. One of the last stages in processing includes precipitation by alcohol, (e.g., ethanol, isopropanol or methanol), and purification [79]. It is known that increased acidity results in increased extraction yields of various types of pectins (e.g., water-soluble, chelator-soluble), and protopectins. This is due to the cleavage of glycosidic bonds in the neutral sugars, as they are more sensitive to pH than the link between two galacturonic acids, resulting therefore in the degradation of the neutral sugar side chains [79]. The extraction technology and strategy will therefore affect the yields, times, costs and the structure of the obtained pectins, which may have different (positive or negative) outcome on functionality. While clearly increased yield is beneficial from industrial aspects and can be achieved by increasing acidity or time, it will result in higher destruction of neutral sugar side chains possibly negatively affecting potential bioactivity that can originate from those groups [56]. Several novel strategies for the extraction of pectins from citrus waste and by-products have been reported [80], including microwave [43,81,82], ultrasound [82,83], high pressure [84,85], subcritical water [86], enzyme utilization [87], electromagnetic induction heating [88] and combination of chelators like citric acid in the conventional process [89]. From the analytical point of view, Fourier transform infrared spectroscopy (FTIR) was found applicable for determining the optimal extraction time for the enzymatic and acidic extraction processes of pectins from lime peel. Authors revealed that this technique could afford prediction of pectin extraction yields and pectin features from measurements on crude pectin extracts [90]. In addition FTIR was also found to be a rapid and accurate method for the determination of the degree of esterification [91], therefore it can be suggested as a promising tool for pectin extraction evaluation.

#### 3.1.1. Ultrasound

The extraction of pectin from grapefruit peels by ultrasound-assisted heating extraction (UAHE) has been studied and compared with conventional extractions. It was reported that UAHE is capable of reducing the extraction time by 37% and temperature by 13 °C. Additionally, it was suggested that the obtained pectin had lower viscosity, molecular weight and degree of esterification, but displayed higher branching [83]. Other authors have reported the optimal conditions for UAHE from grapefruit peel; 0.4 W/mL, 60 °C with a solid-liquid ratio of 1/50 g/m. They concluded that both heat and ultrasound facilitated the extractability, dissolution and degradation of pectin [92]. They also reported a better yield, shorter extraction time and reduced temperature compared to the conventional extraction. Brönsted acidic ionic liquid based ultrasound-microwave synergistic extraction of pectin was recently used for the extraction of pectin from the albedo part of pomelo peels [93]. Authors found a pectin yield of 328.64 mg/g using the optimal extraction conditions, which was significantly higher than yields of conventional methods with reference solvents. Obtained results confirmed that this novel approach is an efficient separation technique for the extraction of pectin from citrus peels.

#### 3.1.2. Microwaves

The use of MAE for only 3 min, compared to two hours with conventional heating, resulted in a slightly improved pectin yield [94]. Another study has reported a significant (up to 250%) yield increase (10 min, 0.63 kW) with improved functional properties [95]. It was also reported that pectin extraction from grapefruit increased the galacturonic acid content (highly responsible for the utilization of pectin as a gelling agent), and esterification degree with the intensification of all MAE parameters, i.e., heating, time and power [82] and that MAE was more efficient than ultrasound assisted extraction. Others previously suggested that this improved MAE yield was caused by a rupture of the cell wall matrix and skin tissues leading to increased interaction between the extracting agent and the plant material [94]. More specifically, it was reported that MAE had the following effects on cell tissue; (i) destruction of parenchymal cells, (ii) changes in specific surface and water absorption, and (iii) inactivation of endogenous enzymes in peels [95].

In another study, the impact of MAE power, time, pH and solid-to-liquid ratio conditions were evaluated and optimized in order to increase pectin yield from orange by-products [81]. As expected, pectin yield increased with increasing microwave power, most likely because of the disruptive effect of microwaves on cell tissue. However, yields dropped with an increase in extraction time, pH and solid-liquid ratio. The authors found the maximum pectin yield was achieved with MAE at P = 422 W, t = 169 s, pH = 1.4 and solid-liquid ratio of 1:16.9 g/mL. Similarly, optimum conditions for pectin extraction from sour orange peel were 700 W, pH = 1.5 and 3 min of irradiation time [96]. The use of an alternative ionic-liquid solvent for pectin extraction from lemon peels has also recently been reported [97] yet no comparison with other extraction methods or solvents was performed.

One interesting study reported that hydrothermal heating of orange peel at lower temperatures with microwave irradiation successfully separated important products, such as pectin, d-Limonene and mesoporous cellulose in one step and without the need for acidity [98]. Microwave heat was also used in an eco-friendly extraction of pectin and d-Limonene from orange waste and lemon peel. The compounds obtained were identified by diffuse reflectance infrared (DRIFT) spectroscopy, while pectinic samples were additionally analysed by electron microscopy. Quality and yield for laboratory vs. semi-industrial production implied good commercialization potential [99]. The combined addition of water, exposure to microwave heating and freeze-drying increased the production of pectin fivefold and showed good potential for industrial applications [49].

#### 3.1.3. High Pressure

High-pressure extraction (HPE), at P = 500 MPa, T = 55 °C, t = 10 min, of orange peel resulted in better yields than conventional extraction and MAE (20.44%, 15.47%, 18.3%, respectively). In addition, the obtained molecular weight and functional properties (e.g., rheological and gelling) were improved, possibly due to the better conservation of the original pectin structure at the lower temperatures used [85] although the effects of pressure on non-covalent interaction could also contribute. A promising combination of enzymatic (cellulose and xylanase) pectin extraction from lime peel under elevated pressures was reported and compared with acidic and aqueous extraction. It was concluded that both pressure (up to 200 MPa) and the enzymes (type and concentration) influenced yield and the degree of esterification in pectin. In addition, those pressures (and enzyme concentrations) had no effect on the molecular weight and viscosity of the product [84]. Yet it is important to note that enzymatic assisted extraction is likely an expensive process and only significant advantage in the received product could make it profitable. Previous works have suggested that pressure (at close to natural pH conditions and high temperature) might result in significant de-esterification of pectin [100,101] and ultra-high pressure homogenization can result in pectin chain degradation and MW decrease [102]. The effects of physical treatments like pressure on the structure and functionality of the obtained product should therefore be tested.

### 3.2. Antioxidant Bioactive Compounds (Polyphenols, Carotenoids, Vitamin E, etc.)

Antioxidant bioactives like polyphenols can be extracted from citrus peel using various methods. Intensity of heat treatment is a common modified parameter that can be used in both pre-extraction and during the extraction itself. One study has documented the influence of drying the peel at different temperatures (50, 60, 70, 80, 90, 100 °C), on content and the antioxidative potential of flavonoids and phenolic acids in *Citrus sinensis* (L.) Osbeck. Temperatures below 60 °C resulted in lower phenolic and flavonoid contents than those above 70 °C, with a peak being around at 100 °C. By contrast, EC50 values, obtained using DPPH and ABTS, were higher at lower temperatures and decreased at higher temperatures, to their lowest value at 100 °C [103]. Shofinita et al. [104] reported a comparison of the quality and amount of antioxidant compounds obtained from the extraction (Soxhlet extraction unit using deionized water) and spray drying of various citrus peels (orange, lemon, lime and mandarin). The average total phenolic content (TPC) of all citrus peel extracts was between 4.9 and 6.9 mg GAE/g fresh weight (FW). Lime peel extract showed the highest antioxidant content (TPC of 6.9 mg GAE/g FW peel and SC50 of 740 μg/mL), but the lowest TPC recovery after spray drying (84%). Lemon and mandarin peel extracts were found to be the most difficult to spray dry (yields/recoveries of 78% and 73%). The differences in composition (citric acid and sugar contents) were suggested as an explanation for the reported differences in spray drying yields.

Extracts obtained by conventional extraction using different solvents from the leaves, cortex and peels of pomelo were studied for the antioxidant capacity and the total phenolic, flavonoid and carotenoid contents. The highest total phenolic content was found in ethyl acetate extracts from the cortex, while highest total flavonoid content was extracted using the same solvent, but from the leaves. For both ethyl acetate extracts, the lowest DPPH scavenging activity was found in the cortex, while the EC_50_ of phosphomolybdenum was lowest for the leaves. The authors singled out flavonoids as the major contributors to the strong antioxidant capacity found in all anatomical pomelo segments [105].

The effect of heating of mandarin (*Citrus unshiu*) peels before conventional extraction was studied by treating the peels at three temperatures (50, 100, 150 °C) and six different times (10, 20, 30, 40, 50, and 60) prior to extraction (70% ethanol, and 0.1 g/10 mL water) followed by measurement of total phenols, radical scavenging activity and reducing power. It was reported that antioxidant activity (total phenolic content, radical scavenging activity (measured by DPPH) and reducing power) of the extracts significantly increased due to heating, as compared to non-heated samples [106]. The outcome of the presented results suggests that heating liberates the antioxidant compounds from the matrix possibly due to a higher extent of matrix destruction, yet it is likely that this effect is superimposed with negative effects of extended thermal treatment on sensitive antioxidant compounds. Therefore, it could be beneficial to increase matrix destruction with minimal negative effect of thermal treatment on the antioxidative compounds, as can be done with several novel processing methods.

#### 3.2.1. Pulsed Electric Fields

The potential of pulsed electric fields (PEF) in the extraction of polyphenols, mainly flavonoids, from orange peels was evaluated, concluding that a significant increase (up to 159%) in polyphenol extraction yield after PEF pre-treatment at an electric field densities of 1 kV/cm and 7 kV/cm (t_PEF_ = 60 μs, 20 pulses, *f* = 1 Hz) can be achieved [107]. It should be noted that a significant increase in polyphenol extraction was observed when electric field strength and treatment time were increased. This fact can be attributed to enhanced cell disintegration, as it is well known that polyphenols are enclosed in intracellular vacuoles. PEF can induce cell permeabilization and favour the formation of pores, thus facilitating the selective extraction of polyphenols. In particular, the recovery yields of naringin and hesperidin increased ≈2- and 3-fold, respectively, as compared to the untreated samples, in samples that were pre-treated with PEF.

#### 3.2.2. Ultrasound

The potential of UAE as a cheap, reproducible and simple alternative to conventional extraction for the recovery of BAC from citrus waste by-products has been reported [108]. The main mechanism makes use of ultrasound’s ability to promote cell disruption by cavitation, thus promoting the acceleration of internal diffusion and increasing mass transfer [108]. For instance, UAE improved polyphenolic extraction from orange peels at 25 kHz/150 W/30 °C/15 min [109] (Table 3). Other authors have reported more intense UAE conditions required for polyphenolic recovery from orange peels [19,110], yet the recovery was still improved compared to conventional methods.

A number of authors have more recently evaluated and optimized ultrasound exposure (38.5 kHz, 50.93 W), solvent type (methanol, ethanol and acetone), and solvent concentration (20%, 50% and 80% *v*/*v*) in the extraction of BACs from mandarin (*Citrus reticulata Blanco* cv. Sainampueng) and lime peels (*Citrus aurantifolia*). Specifically, the BAC obtained included total phenolic compounds, total flavonoids and two flavanone glycosides, hesperidin and naringin. The best results were achieved for mandarin peels, extracted with 80% of acetone, where 3 mg of GAE/100 g DW of total phenolic compounds were obtained, as well as 2.5 mg of quercetin EQ/100 g DW (total flavonoids), and 1.4 mg/100 g DW of hesperidin [111].

Garcia-Castello et al. [112] extracted flavonoid compounds from grapefruit waste using conventional solid-liquid extraction (CE) and ultrasound-assisted extraction (UAE). Naringin was the most abundant flavonoid in the extracts and ranged from 18 to 28 mg/g DW for CE and 24–36 mg/g DW for UAE. UAE was very effective compared to conventional solvent extraction, giving higher extraction yields at lower temperatures and extraction times. They concluded that UAE can furnish economic and environmental advantages [112].

Londono-Londono et al. [19], studied the application of UAE in flavonoid extraction from citrus peels (lime, orange and tangerine). Total phenolic content in the flavonoid fractions, obtained from different sources, was 74.80 ± 1.90, 66.36 ± 0.75 and 58.68 ± 4.01 mg GAE (gallic acid equivalents)/g, for lime, orange and tangerine (*Citrus tangerine*). Orange peel contained hesperidin, neohesperidin, diosmin, nobiletin and tangeritin and was the most complex source. Tangerine peel was the simplest source and contained only hesperidin and neohesperidin. Differences in the antioxidant activity of the individual components of the flavonoid fractions were observed and the yields and total phenolic content from dry material were higher than those in the wet material (*p* < 0.01). Extraction time (within the studied time frame) had no influence on phenolic content. UAE was optimal at a frequency of 60 kHz, extraction time of 30 min and temperature of 40 °C, citrus peel/water ratio (g/mL) 1/10, using Ca(OH)_2_ as the basifying agent and water as the solvent. With these parameters, a yield of 40.25 ± 12.09 mg/g (4.025 ± 1.209% DM) was obtained and the total phenolic content was 19.595 ± 2.114 mg GAE/g of peel dry matter [19].

In another study, Qiao et al. [113], studied the sonochemical effects of ultrasound treatment on 14 flavonoids in common in citrus fruit. Eriocitrin, narirutin, neohesperidin, quercitrin, eridictyol, didymin, naringenin, luteolin, sinensetin, nobiletin, tangeretin, naringin and hesperidin were stable, whereas quercetin degraded significantly by ultrasound treatment. The degradation rate of quercetin was highest in 80% ethanol aqueous solution and decreased with increasing temperature. Quercetin underwent four types of reactions occurring simultaneously under ultrasound treatment; oxidation, addition, polymerization and decomposition. Eight degradation products were identified as the dimer, alcohol addition, oxidation and decomposition products.

Sun et al. [114], evaluated the effects of a number of different factors (particle size, the extraction solvent, solid/solvent ratio, temperature, extraction time, the electrical acoustic intensity, liquid height and duty cycle of ultrasound exposure) on the extraction yield of all-*trans*-β-carotene from citrus peels (Bendizao mandarin (*Citrus succosa Hort*)) by UAE. The extraction yield was significantly affected by particle size. Dichloromethane caused the degradation of all-*trans*-β-carotene extracted during UAE. Ethanol showed significantly higher extraction yields with UAE than with classical extraction. The extraction yield of UAE had a peak value at 25 °C. The extraction yield of UAE decreased with increasing liquid height. The extraction yield increased with increasing duty cycle until equilibrium was achieved [114].

#### 3.2.3. Microwave-Assisted Extraction (MAE)

MAE has been identified as a fast and reliable method for BAC extraction from citrus wastes and by-products and using minimal amounts of solvent (Table 3). For instance, response surface methodology (RSM) was used to evaluate and optimize MAE in polyphenolic recovery from citrus mandarin peels. Moreover, the obtained MAE optimal conditions were compared with conventional rotary extraction (RE) and UAE. The authors obtained the maximum yield at a microwave power of 152 W, extraction time of 49 s, liquid-to-solid ratio of 16 and methanol concentration of 66%. MAE also provided additional advantages over the other methods, judging by the extraction efficiency and antioxidant activity of the obtained extracts [115]. Other studies reported slightly different values of optimal MAE conditions in aqueous [116] and aqueous acetone media [117]. When the utilization of MAE for the extraction of polyphenols from lemon peels was compared with conventional processes and UAE, optimal MAE conditions gave similar total phenolic content (15.78 ± 0.8 mg GAE/g) than optimal UAE conditions (15.22 ± 0.88 mg GAE/g) [118].

A further work evaluated the impact of UAE, MAE, supercritical CO_2_ extraction (SCE), and HPE on total polyphenols and individual flavonoid recovery from orange peels [119]. Authors compared innovative and conventional extractions at a solid-to-liquid ratio of 5:50 *w*/*v* in 80% ethanol under mechanical agitation. The authors identified the following as the optimal conditions for each extraction; UAE at 125 W/35 °C/30 min, MAE at 200 W for 180 s, SCE at 10 MPa and 80 °C and HPE at 50 MPa/35 °C/30 min. However, antioxidant activity was not optimal under these conditions. The highest antioxidant values for MAE and HPE were obtained at 300 W and 100 MPa. Additionally, they concluded that SCE is not very effective, despite its “green” nature, and that this was most likely due to the non-polar nature of subcritical CO_2_.

The same research group has investigated the effect of UAE, MAE and SCE on the extraction of total phenols, total flavonoids, individual flavonoids and antioxidant activity on orange peels and then compared the results to those obtained from conventional extraction. The main flavonoids from orange peel (roughly accounting for 84%), were neohesperidin and hesperidin, and their yield was modified by extraction type. Neohesperidin ranged in quantity from 0.624 ± 0.013 (SCE) to 1.045 ± 0.001 g/100 g orange peel powder for MAE extraction, whereas hesperidin averaged from 0.407 ± 0.008 (SCE) to 0.836 ± 0.029 g/100 g orange peel powder (UAE). 

MAE gave the highest yielding extraction and SCE the lowest. However, conventional extraction was reported to contain the extracts with the highest antioxidant activity [120].

#### 3.2.4. Pressurized Fluid Extraction

A study has reported the influence of the solvent on antioxidant and anti-inflammatory potential in the pressurized fluid extraction (PFE) of mandarin peel extracts (*Citrus unshiu*). The authors studied extracts obtained using ethanol, subcritical and hot water. Subcritical water extraction yielded the highest polyphenolic content, while acidic hydrolysis doubled the polyphenolic and flavonoid content. Acidification accordingly increased the antioxidant activity, as measured by DPPH, of β-carotene and ferric thiocyanate [121].

Another study examined how the alteration of subcritical-water extraction parameters (i.e., time and temperature) modified the total polyphenolic content and antioxidant activity obtained from citrus pomace. The highest values for total polyphenolic content and antioxidant activity (measured by DPPH) were obtained at T = 200 °C, P = 1.4 MPa, t = 60 min. Under these conditions, the authors obtained the highest amounts of polymethoxylated flavones (i.e., sinensetin, nobiletin, and tangeretin) and identified subcritical water extraction as the most appropriate and successful technique for the isolation of antioxidants and nutraceuticals from citrus pomace [122].

#### 3.2.5. High-Pressure Assisted Extraction (HPE)

The air pores in fruit tissues are partially filled with liquid during HPE. Blocked air is freed when the pressure is released, causing cell membrane damage. High-pressure treatment can lead to the deprotonation of charged groups and the disruption of salt-bridges and hydrophobic bonds, resulting in conformational changes and the denaturation of proteins. This makes cellular membranes less selective and renders the compounds more accessible to extraction up to equilibrium [123,124,125,126].

The impact of HPE (300–500 MPa, 3–10 min) on the polyphenolic extraction as well as the antioxidant and antimicrobial activity of citrus peels (lemon, sweet orange) has been reported in two studies [9]. Higher polyphenol extraction yields, as compared to the control samples, from orange and lemon peels were observed when HPE (300 MPa, 10 min; 500 MPa, 3 min) was used. Polyphenol content was significantly higher in the extracts obtained from orange vs. lemon peels, except for at 500 MPa [9]. This can be attributed to the different resistance that orange and lemon peel tissues show to high-pressure treatment. However, when more intense HPE conditions were used (500 MPa, 10 min), phenolic content and total antioxidant activity (by DPPH) decreased for both lemon and orange peels. Moreover, it was observed that the highest polyphenol yield from orange peels was obtained by HPE at 300 MPa for 3 min. Additionally, the obtained orange peel extracts showed effective antimicrobial activity against a wide range of gram-positive and gram-negative bacteria, especially against *Acinetobacter* and *Listeria innocua* [127].

### 3.3. Essential Oils (EOs)

Conventional steam- and hydro-distillation extractions are the most frequently used techniques for the production of EOs on commercial scale [65,128]. When steam distillation and a cold-press extraction, of volatile compounds from Shiikuwasha (*Citrus depressa Hayata*) peels was studied, monoterpene hydrocarbons were the main group of volatiles (~93%) identified by gas chromatography-mass spectrophotometry (GC-MS). They included limonene (~44%; 341.46–379.81 mg/100 g of fresh peel), γ-terpinene (~29%; 219.90–245.86 mg/100 g of fresh peel), and *p*-cymene (~10%; 61.47–97.22 mg/100 g of fresh peel). The cold pressed system retained total phenolics and antioxidative activities in extracts better than steam distillation [129].

Monoterpene hydrocarbons (98.61–99.14%) followed by aldehydes (0.49–0.76%) and alcohols (0.18–0.47%) were the predominate quantitative composition of the volatile part in cold pressed fruit peel essential oils of two cultivars of sweet orange [130]. Previous reports showed that the main components of different varieties of cold-pressed citrus peel oils were limonene (62.5–95.7%), γ-terpinene (0.1–23.3%), α-pinene (0.1–2.5%), and myrcene (1.7–2.0%) [131]. Limonene was the primary ingredient of essential oils of *C. paradisi* (92.83–96.06%) and *C. grandis* (32.63–55.74%) [132]. Limonene was the most abundant in the Japanese (91.8%) and Korean (86.4%) oil. Alcohols accounted for 1.8% in the Korean oil, and 0.2% in the Japanese oil, in which the respective linalool levels were 1.2% and 0.1% [133].

However, the great interest in EO production has driven the development of non-conventional technologies. The improvements mentioned aim to increase yields, reduce operational costs and be compatible with all green-extraction concepts; most importantly, decrease the use of toxic solvents, shorten extraction time, intensify mass transfer, reduce energy consumption and provide high-quality extracts [25,134]. For instance, several innovative approaches have been employed for this purpose, including SCE [135], UAE [136], and MAE [137].

#### 3.3.1. Ultrasound-Assisted Extraction

The use of UAE to improve EO yield has been evaluated. UAE and hydro-distillation (Clevenger method, 26 kHz and 200 W) were combined to extract EOs from orange peels [138]. When using UAE the authors observed a significant reduction in extraction times, as compared to the control samples (without UAE). Mason et al. (2011) found UAE of essential oils from Japanese citrus was increased by 44% when compared to the traditional extraction methods [139]. Essential oil glands are usually present at the surface of the plant, therefore during ultrasonication the collapse of the cavitation bubbles will destroy the glands thus facilitate the mass transfer and the release of plant essential oil [140]. The enhancement of cavitational activity is strongly dependent on the operating conditions such as the frequency, pressure amplitude, temperature, the length of treatment time, etc. However, essential oil may be prone to oxidation after prolonged utilization of sonotrode due to metallic contamination [141].

#### 3.3.2. Microwaves

Microwave has been useful to improve EO extraction from citrus waste and by-products. It was found to improve the recovery and quality of d-limonene from orange peels, as compared to conventional heating, and did so in shorter periods of time [142]. The authors attributed these advantages to the ability of MAE to promote cellular disruption, thus favouring the release of limonene from the plant tissues. In both cases, significant improvements in the yield and quality of d-limonene were shown, as compared with conventional methods, thus proving the usefulness of MAE in industrial applications. In a study by Bustamante et al. [143], microwave-assisted hydro-distillation (MAHD) was used to extract the essential oil present in wet citrus peel waste. Optimal conditions for essential oil MAHD involved the irradiation of a waste orange peel:water mixture (1:1.5) over two subsequent steps using a variety of irradiation powers for a total extraction time of 20 min (at a constant pressure of 300 mbar throughout the process). The essential oil yield obtained from oranges, using MAHD, was 1.8 ± 0.1% (dry basis) and was comparable to the oil obtained from conventional hydro-distillation (1.7 ± 0.1%; dry basis). It was shown that MAHD allows for the fast and reproducible production of essential oil and reduces energy and solvent consumption, as compared to conventional methods [143].

#### 3.3.3. Supercritical Fluid Extraction

Atti-Santos et al. [144] have evaluated the use of combined hydrodistillation + supercritical carbon dioxide (SC-CO_2_) on the extraction of essential oils from lime (*Citrus latifolia* Tanaka). For this purpose, they evaluated a number of SC-CO_2_ processing parameters (temperature, pressure, CO_2_ flow, extraction time and material characteristics). They observed the optimal conditions for the recovery from lime peels when they used SC-CO_2_ (90 bar, 60 °C, CO_2_ flow rate of 1 mL/min, 30 min) on milled peels. They also observed the best lime oil yields after hydrodistillation (5.45% *w*/*w*) and supercritical extraction (7.93% *w*/*w*) on milled peels. Others evaluated the impact of SC-CO_2_ (10–30 Mpa, 40–80 °C) on the extraction of essential oil from Kabosu (*Citrus sphaerocarpa* Tanaka) peel [145]. The authors obtained the maximum essential oil yield (1.55% by weight of wet sample) after SC-CO_2_ (20 MPa, 80 °C). This yield was 13 times higher than under the conventional cold-press method. The authors identified 49 compounds (non-polar and weakly polar hydrocarbons, such as terpenoid, free fatty acid and coumarin) using GC/MS. SC-CO_2_ extracts presented lower monoterpene content and higher oxygenated compound content of sesquiterpenes, as compared to conventional extraction. Moreover, a bioactive compound (auraptene) was obtained in the SC-CO_2_ extract.

On the other hand, Chen and Huang [146] have used SC-CO_2_ technology (20 MPa, 50 °C, CO_2_ flow rate of 6 mL/min) to extract oleoresin from the peels of three citrus varieties Ponkan (*C. reticulate* Blanco), Tankan (*C. tankan* Hayata), Murcott (*C. reticulate × C. sinensis*)) by adding alcohol as a solvent assistant to enhance the extraction rate. These authors noticed that the non-volatile oleoresin extracted from the samples contained polymethoxyflavones (between 86.2 and 259.5 mg/g), limonoids (between 111.7 and 406.2 mg/g), and phytosterols (between 686.1 and1316.4 µg/g). In addition, He et al. [147], have also studied the SC-CO_2_ (39 MPa, 80 °C) extraction of flavonoids from pomelo peel and their antioxidant activity. They concluded that it gave higher flavonoid extraction yields under lower extraction times, giving better scavenging activities of hydroxyl, DPPH and ABTS radicals, than conventional solvent extraction.

SC-CO_2_ (200 and 250 bars, 45 and 60 °C) and conventional hexane extraction (70 °C) were used to extract oils from a mixture of citrus seeds and peels [148]. These authors found an enhanced oil recovery when hexane extraction was used. Moreover, the oils were more stable from an oxidative point of view compared to SC-CO2 oils. However, it should be noted that the oils obtained after SC-CO_2_ presented better antioxidant and antimicrobial properties.

#### 3.3.4. Enzyme-Assisted Extraction

Recent study employing cellulose enzyme pre-treatment for the extraction of essential oils from three different citrus peels [149]. Compared to conventional methods, the use of enzyme-assisted extraction resulting in an increased yield of essential oil from two to six times for orange and grapefruit peel, respectively. The major action of cellulase and hemicellulase include hydrolysis of cell walls components thus the permeability of the cell wall increases leading to higher yields. Also, enzyme treatment reduced viscosity and aiding in the breaking of an emulsion to recover oil from the aqueous phase [150]. Extraction of essential oils from the flavedo of mandarin peels by Xylananses enzyme pre-treatment (varying concentration from 0.1%, 0.2% and 0.3%), followed by hydrodistillation and cold pressing extraction has been studied. Obtained results showed the increase in the yield of essential oil compared to the control sample by up to 15% [151]. This improved extraction efficiency can be attributed to rupturing of the oil sacs/glands by the enzymatic action leading to excess release of oil from oil sacs.

### 3.4. Anaerobic Digestion

In addition to the major possible utilization for BACs extraction the citrus waste (CW) contains various water soluble and insoluble carbohydrates that are an excellent raw material for conversion into biological biofuels, such as ethanol and biogas. The results show that wastes produced from citrus have good potential for biofuel production. The ethanol and biogas produced from CW is estimated to be 26.98 million litres and 37.08 million m^3^ (in Iran), respectively [152]. Boluda-Aguilar et al. [153], have studied the application of steam explosion and enzymatic hydrolysis pretreatment on lemon (*Citrus limón* L.) citrus peel wastes. They obtained bioethanol, galacturonic acid and other co-products, such as d-limonene and citrus pulp pellets. The steam-exploded lemon peel wastes were sequentially processed by hydrolysis and fermentation. The steam explosion pre-treatment reduced the residual content of essential oils below 0.025% and significantly decreased hydrolytic enzyme requirements. Ethanol production in excess of 60 L/1000 kg fresh lemon peel biomass was obtained [153]. Another study involved slow pyrolysis (200–650 °C) experiments on citrus residues (orange peel waste and lemon peel waste). Thermogravimetric analysis (TGA) highlighted the higher stability of lemon peel waste, which appears to be related to the higher lignin content. Torrefaction (a thermal process used to produce better fuel characteristics than the original biomass) of lemon and orange residues, in the temperature range of 200–325 °C, produced fuel with increased energy density and stability [154]. The utilization of CW for biological fuels can and should be considered as a step after possible BACs extraction. Negro et al. [155], recently reviewed the extraction of d-limonene from citrus peels and the residue from fruit juice production process. They concluded that anaerobic digestion and fermentation are suitable post-limonene extraction processes [155]. In another study, Ruiz et al. [156], have studied anaerobic digestion process inhibition by limonene, the main component of citrus essential oils present in citrus peel. The biochemical methane potential (BMP) values of the citrus waste tested (orange peel, mandarin peel, mandarin pulp and rotten fruit) were 354–398 L_CH4_ kg_vs_^−1^. The methane course and IC_50_ values indicate that reversible inhibition and biomass activity recovery occur during the anaerobic digestion process, despite the non-reversible antimicrobial mechanism described in the literature for limonene. They also found that the inhibition of the anaerobic digestion process by limonene was reversible and that the IC_50_ value increased to 669 mg∙kg^−1^ for a second load of limonene, indicating that some biomass adaptation may occur [156]. Koppar et al. studied the impact of citrus waste on methane production in a batch anaerobic digester that was operated at a thermophilic (55 °C) temperature for the biogasification of citrus (orange) peel waste for 25 days [157]. An energy analysis showed that the biogas produced from the waste streams of a citrus processing plant handling 600 tons of fruit per day is more than sufficient to meet all its electricity and fuel demands [157]. Ruiz & Flotats [158], have studied the valorization possibilities of citrus waste and concluded that anaerobic digestion for methane production appears as the most technically feasible and environmentally friendly alternative. However, citrus essential oils can inhibit such biological process. Therefore, successful strategies to avoid process inhibition by citrus essential oils are based on either the recovery or removal of limonene, by extraction or fungal pre-treatment, respectively [158].

## 4. Challenges and Perspectives

**Figure 3:**
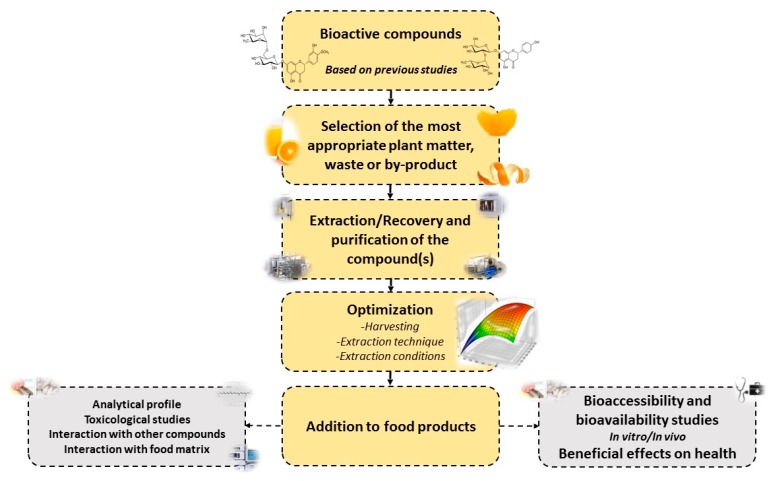
Set-up for the development and evaluation of a new functional bioactive compound- (extracts and/or isolated compounds) based product.

Citrus waste is a good source of bioactive compounds often discarded but can be used as food additives and/or nutraceuticals. The use of BACs such as common antioxidant compounds from citrus waste for the development of new functional products or nutraceuticals lies on the border between pharmacy and health and presents a growing interest. A diagrammatic representation of the main steps to be followed when developing new food products that are based on BACs from citrus waste and by-products is shown in Figure 3. 

First of all, the appropriate optimal source for the targeted bioactive compound is required, as the content of different compounds greatly differs between the citrus species as well as their location in the fruit tissue. While the bioactive compounds often present in the fruit itself and the processing by-products, in many cases, fitting well with the goal of waste reduction, such compounds can be in high content in the discarded parts. Once the material has been selected, the extraction techniques and conditions have to be optimized for each matrix and compound. In this respect, it is not only the extracted amount of compounds that is important, but also the profile. Sometimes, a purification step may be required to obtain pure compounds for pharma or food production, yet such steps can result in the utilization of non-green solvents and a significant increase in costs. Once the extracts, or the isolated compounds, are added into the products, it is necessary to evaluate the bioaccessibility and bioavailability of these compounds, as well as the interaction between the compounds and other components found in the matrix, which can modify the availability of these molecules.

When considering waste utilization, always many aspects must be taken into account, and while the described green methods are novel, promising and can reduce negative environmental problems, they can result in significant increase in costs, which may not worth the added value in yield or purity from a commercial point of view. Therefore, all strategies should be evaluated taking into account all aspects including legislative considerations that can drive greener technologies. A possible utilization of the CW in addition to or even replacing the BACs extraction, could be the formation of bio-fuel. Off course if after a step of green extraction of compounds from the CW the residual used waste is utilized for energy production, such process combination could present a promising and green approach.

## Figures and Tables

**Table 1:** Fibre composition (% dry weight) of different *citrus fruit* by-products. Adapted from Marín et al. [3].

*Citrus* Waste	Pectin	Lignin	Cellulose	Hemicellulose
Lemon peels	13.00 ± 1.06	7.56 ± 0.54	23.06 ± 2.11	8.09 ± 0.81
Lemon pulp	22.53 ± 1.95	7.55 ± 0.66	36.22 ± 3.24	11.05 ± 1.09
Orange peels	23.02 ± 2.12	7.52 ± 0.59	37.08 ± 3.1	11.04 ± 1.05
Orange pulp	12.07 ± 1.12	7.51 ± 0.62	24.52 ± 2.0	7.57 ± 0.66

**Table 2:** Structures and types of carotenoids derived from *citrus* peel.

Type	Basic Structure
α-Carotene	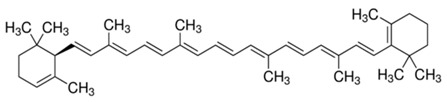
β-Carotene	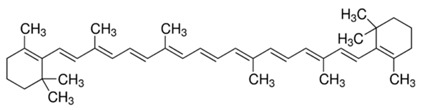
Lutein	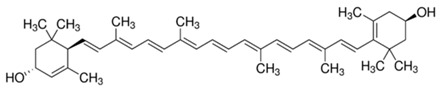
Zeaxanthin	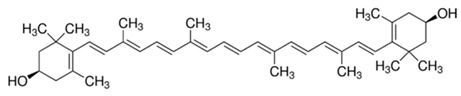
β-Cryptoxanthin	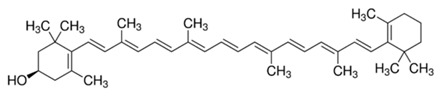

**Table 3:** Effect of ultrasound-assisted extraction and microwave-assisted extraction on antioxidant bioactive compound extraction from plant materials compared to conventional extraction. Adapted from Roselló-Soto et al. [108].

Plant Material									
Ultrasound-assisted extraction									
	Treatment conditions						Ethanol/Water Ratio (*v*/*v*)	Extraction yield	Reference
	kHz	W		°C		min			
Orange peel	25	150		30		15	50:50	Polyphenols (caffeic (207%), *p*-coumaric (180%), ferulic (192%), sinapic acid (66%), *p*-hydroxybenzoic (94%))	[110]
	25	50–150		10–40		60	20–80:80–20	Polyphenols (naringin (38%), Hesperidin (42%), total phenolic compounds (31%))	[111]
	-	125		35		30	80:20	-	[120]
Microwave-assisted extraction									
	Treatment conditions							Extraction yield	
	W		°C		s		Liquid-to solid ratio		
Orange peels	500		<135		122		25 mL·g^−1^	Polyphenol content (12.20 mg/GAE g^−1^ DW)	[118]
	200		-		180		-	-	[120]
Lemon peels	400		123				28:1 mL	Polyphenol content (15.74 mg/GAE g^−1^ DW)	[119]
Mandarin peels	400		<135		180		1:2	-	[117]
	152				49		16	-	[116]
